# The Assessment of Strength of Cementitious Materials Impregnated Using Hydrophobic Agents Based on Near-Surface Hardness Measurements

**DOI:** 10.3390/ma14164583

**Published:** 2021-08-15

**Authors:** Martyna Nieświec, Łukasz Sadowski

**Affiliations:** Department of Materials Engineering and Construction Processes, Wroclaw University of Science and Technology, Wybrzeże Wyspiańskiego 27, 50-370 Wroclaw, Poland; martynalewek@gmail.com

**Keywords:** impregnation, hydrophobic agent, cementitious materials, near-surface hardness

## Abstract

Recently, the surfaces of concrete structures are impregnated to protect them against the environment in order to increase their durability. It is still not known how the use of these agents affects the near-surface hardness of concrete. This is especially important for experts who use the near-surface hardness of concrete for estimating its compressive strength. The impregnation agents are colorless and, thus, without knowledge of their use, mistakes can be made when testing the surface hardness of concrete. This paper presents the results of investigations concerning the impact of impregnation on the subsurface hardness concrete measured using a Schmidt hammer. For this research, samples of cement paste with a water–cement ratio of 0.4 and 0.5 were used. The samples were impregnated with one, two, and three layers of two different agents. The first agent has been made based on silanes and siloxanes and the second agent has been made based on based on polymers. The obtained research results allow for the conclusion that impregnation affects the near-surface hardness of concrete. This research highlights the fact that a lack of knowledge about the applied impregnation of concrete when testing its near-surface hardness, which is then translated into its compressive strength, can lead to serious mistakes.

## 1. Introduction

Among the available diagnostic methods intended for reinforced concrete structures, the non-destructive techniques, that do not affect the structure of the tested elements are very useful. The requirements concerning these methods are still changing, and therefore further development of techniques and devices for non-destructive testing is necessary. There is also a growing demand for educated personnel with a high level of knowledge, and therefore education in this field is becoming very important. Currently, non-destructive testing techniques are the subject of many scientific and technical conferences, and are used to diagnose civil engineering structures [1,2,3,4]. Among them you can find both traditional methods, e.g., ultrasonic, penetration, or sclerometric methods, as well as laser, radar, or optical techniques allowing for determination of the deformation of an element [5,6].

One well-known and recognized test among non-destructive methods is the near-surface hardness measurement [7]. It enables the near-surface concrete hardness to be testing (while leaving a small indentation in the structure), and allows for the obtained values to be correlated with concrete strength [1]. A hammer strikes a ball or indenter with a known energy, and the height to which it recoils is proportional to the hardness of the concrete. The test procedure is described in the standard [8], which states that it can be used to assess the uniformity of concrete in a structure, as well as to determine the areas and parts of a structure where the concrete has deteriorated or is of poor quality. It should be remembered that this procedure cannot be considered as an alternative for determining the compressive strength of concrete, but with a proper correlation, it may allow this value to be estimated [9].

Near-surface hardness measurements in addition to ultrasonic testing [10] can also be used for the preliminary assessment of concrete quality in the near-surface zone after a fire or in heat exposed concrete [11]. Moreover, it helps to determine whether a structure at a given location has been damaged, is slightly damaged, or has not been damaged [12]. Filipowicz et al. [13] checked the suitability of near-surface hardness measurement for architectural examinations of brick walls, but this method turned out to be rather useless. In order to test brick walls with a near-surface hardness measurement, the test method must be carefully elaborated and adapted. Also, in works [14,15,16] the use of a near-surface hardness measurement to assess the uniformity of the condition of bricks and mortar, verify repairs, the degree of degradation and compressive strength of brick, mortars, and walls has been presented.

The results of sclerometric tests are influenced by many factors, as described in papers [17,18,19]. These include, for example, the surface moisture content, the carbonation of concrete, the ambient temperature, the type of surface tested, the age of concrete at the time of testing, the morphology of concrete, and the calibration of the hammer [20,21]. Testing should not be performed in areas where the concrete has carbonated, or where there are cracks, cement slurry accumulations, or coarse aggregates. When testing existing structures, the places where carbonation is least aqueous should be selected. The same applies to frozen or wet areas. Ice caused by freezing of unbound water in the concrete will cause the rebound number to be overestimated, while moisture in the surface will cause it to be underestimated. For this reason, the standard PN-EN 12504-2:2002 [8] recommends using near-surface hardness measurements in the temperature range of +10 to +30 °C [22]. The most important factors influencing the study are listed in Table 1. Concrete is a heterogeneous material, and therefore local non-uniformity may distort test results. It should be noted that past studies did not analyze the effect of the impregnation of concrete using hydrophobic agents, which is the novelty in the research conducted in this work.

Performing near-surface hardness measurements is not complicated. It is more difficult to correlate the obtained results, followed by their translation into concrete strength. Scaling curves that allow compression strength calculations from correlated equations can be found in PN-EN 13791:2008 [27] and in ITB 210/77 [28]. Plechawski compared the scaling methods of correlation curves using the example of a reinforced concrete slab-and-rib floor [29]. His research shows that the average compressive strength calculated according to the curve from the ITB is more similar to the values obtained from core samples than the strength calculated according to the curve from the PN-EN 13791:2008 [27] standard. An individual correlation curve should be created for each study. Paper [1] shows how the choosing of an inappropriate curve leads to errors.

The European Union requires the unification of rules for the use of non-destructive methods [30] but the standard [27] allows the use of correlation equations other than those contained therein. Manufacturers of the devices also provide correlation curves that are located on hammers. In work [1], the curves of hammers (provided by the manufacturer) were compared with the curves that were obtained by the authors. The strength calculated on from the fact that the research material may not match the material that was used for the calibration [26].

Impregnation with hydrophobic agents are, according to the standard [31], on of the three surface protection treatments. Hydrophobic impregnation is most often based on silanes or siloxanes, small particles, thanks to which it is able to easily penetrate the pores and reduce surface tension concrete [32]. First, the silane hydrolysis reacts with water or water vapor, and the silanol molecules then condense into silicone, which react with the hydroxyl group. The silicone resin then bonds to the substrate during drying and creates a water-repellent effect [33]. According to [34], the application of the hydrophobic agents causes an increase in the contact angle. Impregnation using hydrophobic agents is a way to protect the material from environmental influences and to increase its durability. Moreover, it also has a positive effect on the material’s resistance to salt scale deposits [35]. As shown by the research carried out in [35], the influence of hydrophobic agents on the mechanical properties of concrete is insignificant. In [36] examined the feasibility of using hydrophobic agents for surface protection of lightweight concrete modified with waste polystyrene.

Elements that are subjected to environmental influences, e.g., concrete bridges, are most often impregnated using hydrophobic agents. There are colored (in several colors such as yellow, green, etc.) available on the market, but more often colorless hydrophobic agents are used to preserve the natural appearance of structures. It is still not known how the use of hydrophobic agents affects the near-surface hardness of concrete. This is especially important for experts who use the near-surface hardness of concrete for estimating its compressive strength. The impregnation agents are colorless, and thus without knowledge of their use, mistakes can be made when testing the surface hardness of concrete.

Considering the above, this paper presents the results of investigations concerning the impact of impregnation on the subsurface hardness concrete measured using a Schmidt hammer. For this research samples of cement paste with a water–cement ratio of 0.4 and 0.5 were used. The samples were impregnated with one, two, and three layers of two different agents. First agent has been made based on silanes and siloxanes and the second agent has been made based on based on polymers.

## 2. Materials and Methods

### 2.1. Preparation of Concrete Samples

**Figure 1:**
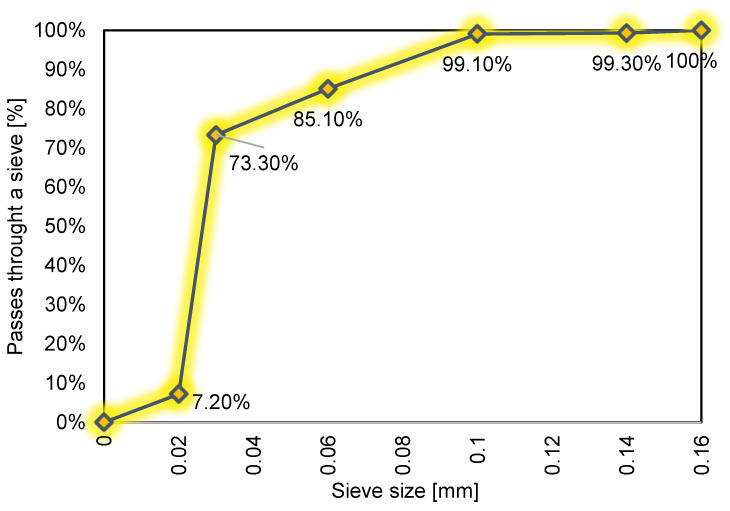
Particle size distribution of cement used to prepare the samples.

In this study, the effect of impregnating concrete (with the use of hydrophobic agents) on its near-surface hardness (assessed using near-surface hardness measurements) was investigated. For this purpose, 18 cubic samples of cement paste with a side length of 10 cm were made—9 with a water–cement ratio of 0.4, and 9 with a water–cement ratio of 0.5. The composition of the concrete mix is shown in Table 2 and particle size distribution in Figure 1. The samples with the ratio *w*/*c* = 0.4 obtained the compressive strength estimated equal to 45.15 MPa, in turn samples with a *w/c* ratio of 0.5 33.35 MPa. The samples were prepared from cement paste due to the fact aggregate affects the results of near-surface hardness measurements. Portland cement CEM I 42.5 from Górażdże (Górażdże, Poland) was used to make the samples which consists of Portland clinker in the amount of 95–100%, secondary components in the amount of <5%, and tap water.

### 2.2. Impregnation of the Concrete Samples Using Hydrophobic Agents

After demolding, the samples were allowed to mature at room temperature for 28 days in 100% humidity. Fourteen visually superior samples were then selected and prepared for impregnation using hydrophobic agents according to the manufacturer’s recommendations. The impregnated surface was first cleaned with a damp cloth and then with a dry cloth and brush. Two impregnations were made using the following hydrophobic agents:

A—based on silanes and siloxanes with a density of 1 g/cm^3^. The product has a low-molecular structure, and therefore has a high penetration capacity, does not clog pores, does not change the appearance of the impregnated surface, protects against dirt, and prevents efflorescence.

B—based on a dispersion of polymers in water. It improves the resistance of the impregnated surface to the influence of weather conditions and UV radiation. When used with concrete tiles, it causes a “wet tile” effect, but does not cause shine. The hydrophobic agent is white, colorless after drying, and the total drying time is 8 h. The required interval between layers is 6 min.

The hydrophobic agent was applied evenly by brush—one, two, and three coats, respectively, for the given samples in short intervals following the manufacturer’s recommended “wet on wet” principle. None of the hydrophobic agents caused any change in the appearance of the impregnated surface. The dosage was 250 mL/m^2^.

Thus, fourteen samples were prepared for further study, the details of which are presented in Table 3. The used designation of samples refers to: the first number—*w/c* ratio; (4—ratio 0.4; 5—ratio 0.5), the second number—number of impregnation layers, and the letter—type of hydrophobic agent.

### 2.3. Testing of Near-Surface Hardness Using the Sclerometric Method

The tests were carried out using a Schmidt’s hammer type N-Proceq, Schwerzenbach, Switzerland. The tests were carried out in accordance with the standard [24]. This is the most common type of test for testing reinforced concrete structures. The test is conducted by holding the hammer firmly perpendicular to the surface, and gradually increasing the pressure until the hammer strikes, after which the number of rebounds is recorded. Adjacent test points should be no closer than 25 mm to both each other and to the edges. If the test results in crushing or damage to the concrete at the measurement point, the result should be rejected [1]. The hammer was checked on a calibration steel anvil prior to testing—6 control measurements were taken, each indicating a rebound number of 80 ± 2.

**Figure 2:**
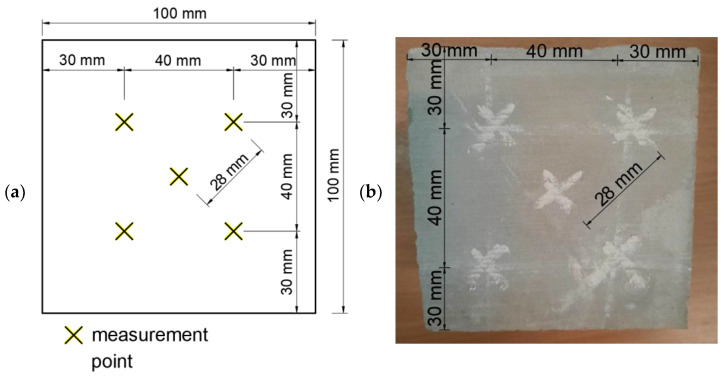
View of the samples with marked measuring points: (**a**) distances shown in the diagram and (**b**) distances marked on a sample.

Measurements were then made on the four sides of all the specimens. Most of the measurements were carried out at five measurement points, the distribution of which is shown in Figure 2. The tests were carried out so that the distance between the measurement points was a minimum of 25 mm, as was the case with the distance from measuring points and the edge. During all the measurements, the hammer was positioned at 90° to the specimen’s surface. The research was conducted in greater compliance with the standard [24] than the other standard [8].

## 3. Results and Analysis

**Figure 3:**
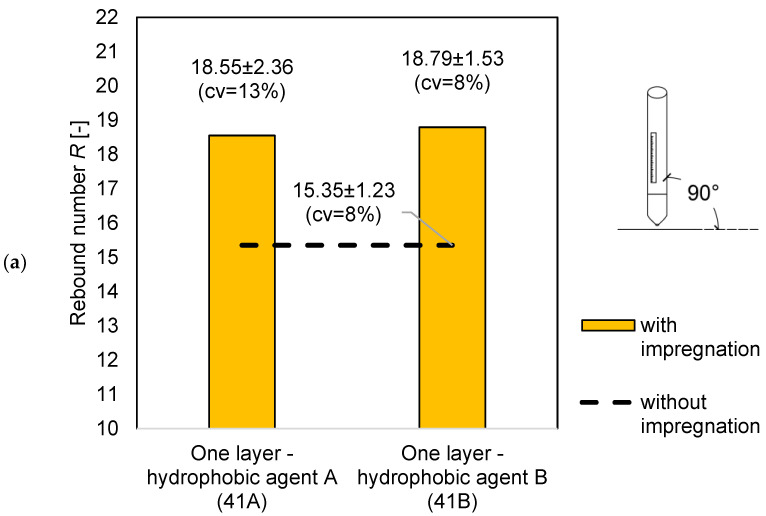

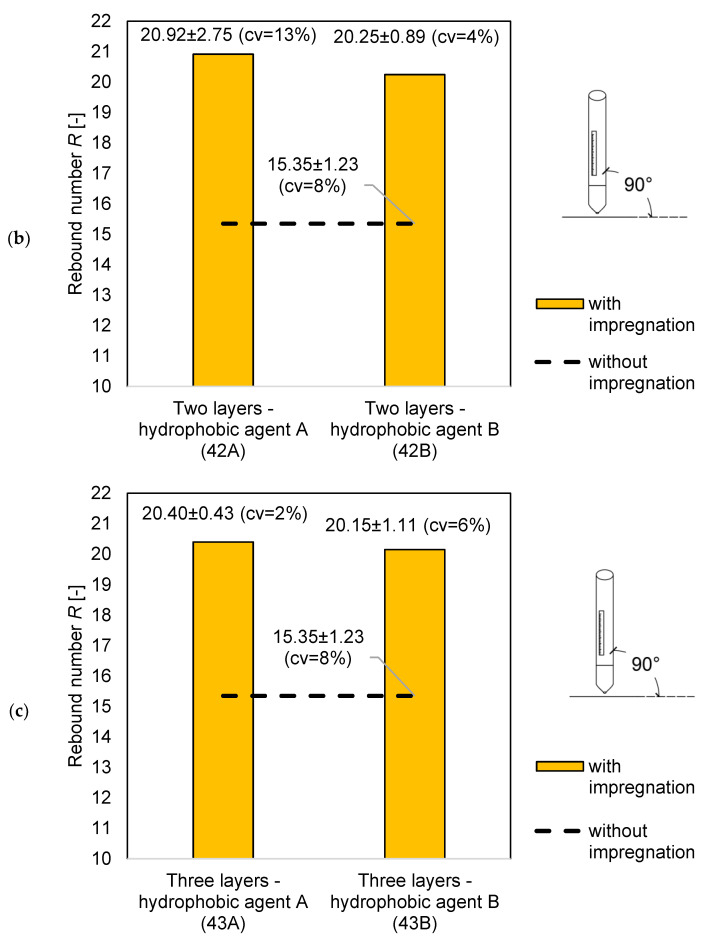
Comparison of the average rebound number for the samples with *w/c* = 0.4: (**a**) without impregnation and with one layer of the hydrophobic agent; (**b**) without impregnation and with two layers of the hydrophobic agent; and (**c**) without impregnation and with three layers of the hydrophobic agent.

**Figure 4:**
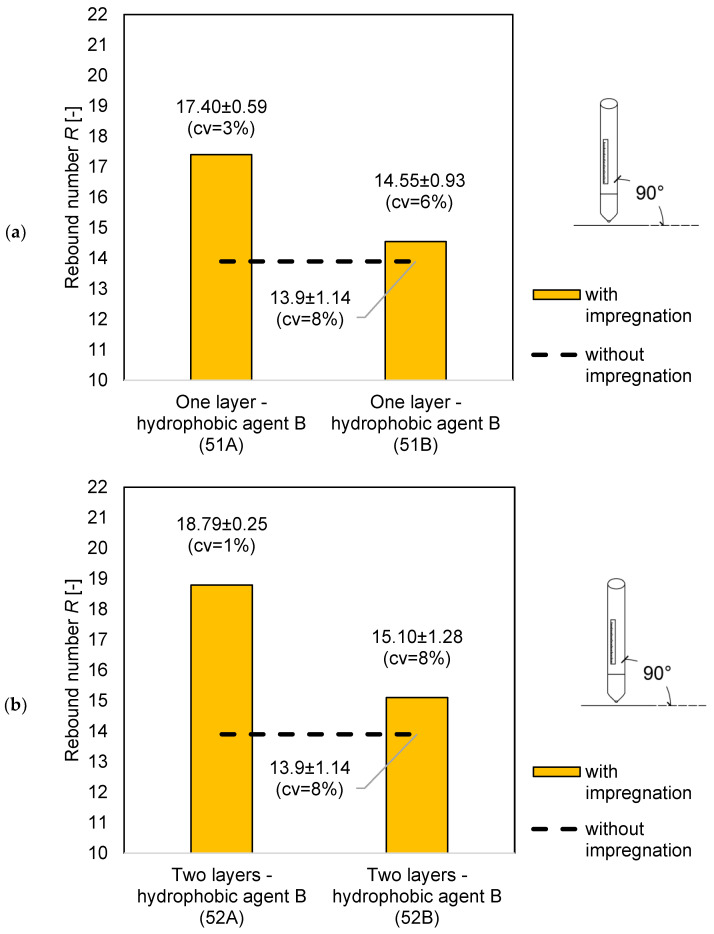

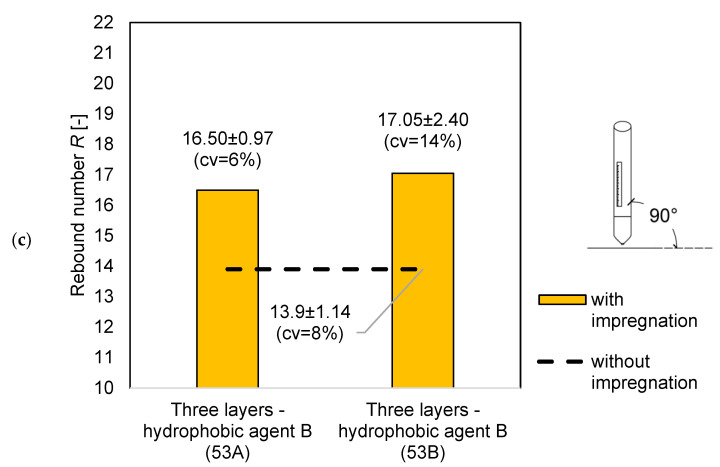
Comparison of the average rebound number for the samples with *w/c* = 0.5: (**a**) without impregnation and with one layer of the hydrophobic agent; (**b**) without impregnation and with two layers of the hydrophobic agent; and (**c**) without impregnation and with three layers of the hydrophobic agent.

For the measurements on the samples with *w/c* = 0.4, all of the impregnated samples had a higher rebound number value than the samples without impregnation. The rebound number increased with the number of impregnation layers. The highest increase in the rebound number can be seen between the samples without impregnation and the samples with one impregnation layer. There is no increase in the rebound number between the samples with two and three impregnation layers, as can be seen in the graphs shown in Figure 3 and Figure 4.

The graphs above show that the impregnated samples have a higher rebound number value. For one impregnation layer, the samples have almost equal values. When the use of hydrophobic agent A, the rebound number increased by 5.57 units, while the use of hydrophobic agent B by 4.9, which is a difference of over 20% compared to the sample without impregnation.

For the samples with the *w/c* = 0.5 with one and two impregnation layers, higher rebound numbers were obtained for the samples with hydrophobic agent A. For three impregnation layers, the sample with hydrophobic agent B obtained a higher value.

Figure 4b,c shows a greater spread of errors, however, according to standard [8], the results can be considered correct. According to standard [8] the measuring point in which 20% of the readings differ from the mean value by more than six units should be rejected. In the case of the conducted research, there difference was no greater than six units in any of the measuring point.

The results of the average rebound number for the specimens with *w/c* = 0.4 are shown in the graph in Figure 4. A similar relationship can be seen here—all the impregnated specimens have a higher rebound number value than the samples without impregnation. Only the samples with hydrophobic agent B and a *w/c* = 0.5 show an increasing dependence with an increase in the number of layers of the hydrophobic agent, while the samples with hydrophobic agent A do not show this dependence. When the use of hydrophobic agent A, the rebound number increased by 4.89 units, while the use of hydrophobic agent B by 3.15, which is a difference of over 20% compared to the sample without impregnation.

**Figure 5:**
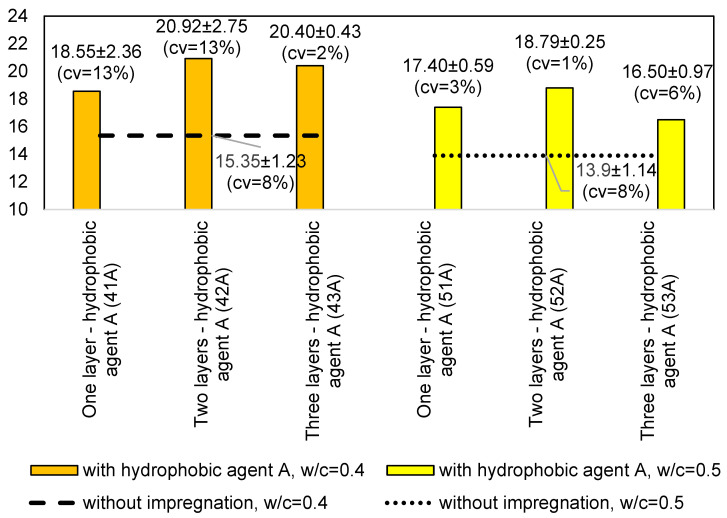
Rebound number comparison for samples with different *w/c* and impregnation using hydrophobic agent A.

Samples with ratio *w/c* = 0.4 have higher variability than samples with ratio *w/c* = 0.5, as seen on Figure 5. Only after applying three layers does it drop to a similar level. It is visible that hydrophobic agent A works better on porous surfaces, therefore for ratio *w/c* = 0.5 the variability is quite low.

**Figure 6:**
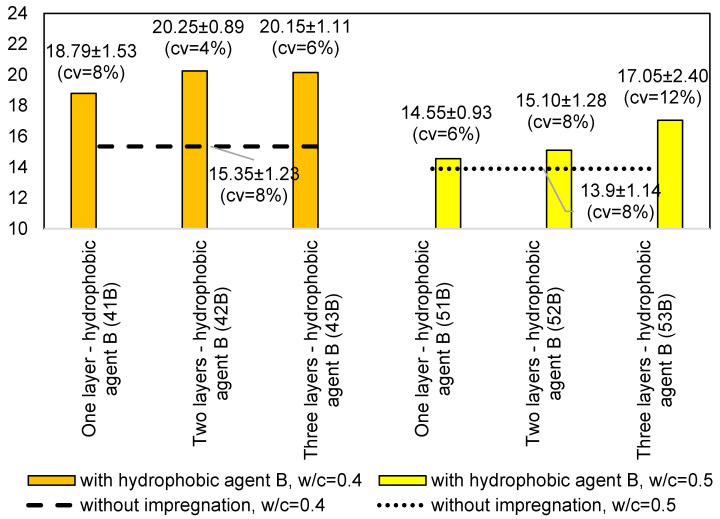
Rebound number comparison for samples with different *w/c* and impregnation using hydrophobic agent B.

Rebound number comparison for samples impregnation using hydrophobic agent B presents Figure 6. For hydrophobic agent A the use of more than two layers does not increase the rebound number. Hydrophobic agent B is more effective for samples with a higher *w/c* ratio. It is visible that with increasing number of impregnation layers, the hardness also increase.

As is well known, the water–cement ratio has a significant effect on the rebound number obtained during Schmidt hammer tests. This is related to the effect of the *w/c* ratio on concrete strength—the lower the water–cement ratio, the higher the compressive strength of concrete and the higher the rebound number.

To illustrate how concrete impregnation can affect the estimation of concrete compressive strength, the ITB scaling curve equation was used:

*f_R_* = 7.4 − 0.915*R* + 0.041*R*^2^ (1)

where: *f_R_*-estimated compressive strength (MPa) and *R*-rebound number (-).

**Figure 7:**
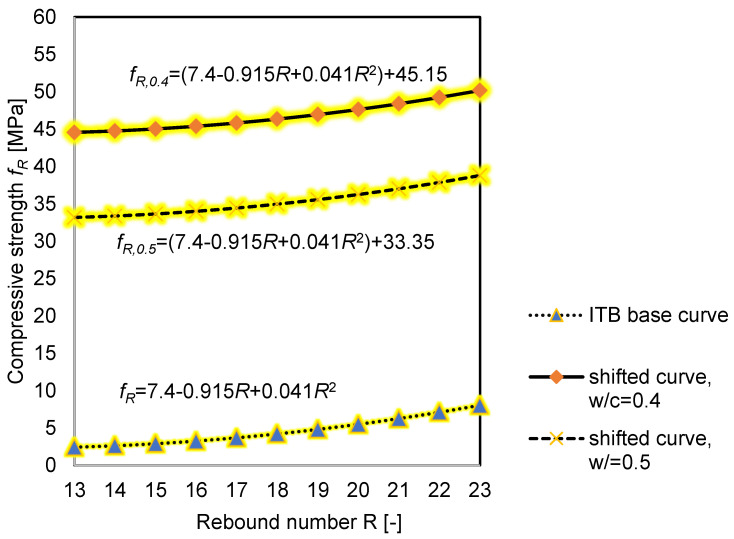
Curve dependence of concrete strength on the rebound number *f_R_-R.*

Figure 7 shows the base curve according to ITB [28], and the shifted curves for the *w/c* ratio 0.4 and 0.5. On their basis, the estimated compressive strength is presented in Table 4. The curves were shifted by the value of the compressive strength obtained from the reference samples and are described below in Equations (2) and (3):

*f_R,0.4_* = (7.4 − 0.915*R* + 0.041*R*^2^) + 45.15 (2)

*f_R,0.5_* = (7.4 − 0.915*R* + 0.041*R*^2^) + 33.35 (3)

It can be concluded from Table 4, that for the samples with a lower *w/c* ratio, greater differences in the results (of more than 3 MPa) can be noticed, which gives a 7% error. For the *w/c* = 0.5, the greatest difference is 6%.

## 4. Conclusions

This paper presents the results of investigations concerning the impact of impregnation on the subsurface hardness concrete measured using a Schmidt hammer. For this research, samples of cement paste with a water–cement ratio of 0.4 and 0.5 were used. The samples were impregnated with one, two, and three layers of two different agents. First agent (A) has been made based on silanes and siloxanes and the second agent (B) has been made based on based on polymers. The research conducted in this study showed that:

- the impregnation of concrete using hydrophobic agents affects its near-surface hardness, and consequently the results obtained in the correlation of the rebound number and its relation to the compressive strength of concrete;
- all of the samples impregnated using hydrophobic agents have a higher rebound number than the samples without impregnation;
- the rebound number increases with the number of impregnation layers. The highest increase in the rebound number can be seen between the samples without impregnation and the first impregnation layer. When the use for samples with ratio *w/c* = 0.4 of hydrophobic agent A, the rebound number increased by 5.57 units, while the use of hydrophobic agent B by 4.9. When the use for samples with ratio *w/c* = 0.5 of hydrophobic agent A, the rebound number increased by 4.89 units, while the use of hydrophobic agent B by 3.15. In both cases, the difference is over 20%;
- based on conducted research it is not possible to clearly state which hydrophobic agent allowed a higher rebound number to be obtained;
- samples with ratio *w/c* = 0.4 have higher variability than samples with ratio *w/c* = 0.5. Only after applying three layers does it drop to a similar level. Hydrophobic agent A works better on porous surfaces, therefore for ratio *w/c* = 0.5 the variability is quite low;
- for hydrophobic agent A the use of more than two layers does not increase the number of rebound. Hydrophobic agent B is more effective for samples with a higher *w/c* ratio. You can see that the more impregnation layers, the higher the hardness, differences between the impregnated and non-impregnated samples when estimating the compressive strength of concrete are up to 7%;
- after exceeding the specified impregnation thickness, there is no significant difference between the number of impregnation layers used; and
- during the tests, higher values of the rebound number were obtained for the samples with a lower *w/c* ratio.

This research highlights an important fact that while testing the concrete with a near-surface hardness measurement and not knowing about the applied impregnation using hydrophobic agents, a big mistake can be made in transformation of the obtained values into the strength of concrete. With regards to further research in this direction, it would be worth investigating the influence of more layers and using a sample with different characteristics. It is also desirable to check other hydrophobic agent and to look at the microstructure of the samples.

## Figures and Tables

**Table 1:** Known factors influencing near-surface hardness measurement.

Name of the Factor	Description
Age of concrete	Is associated with the carbonation of concrete, which causes an uneven distribution of strength. The greatest impact of carbonation occurs in the first year. Near-surface hardness measurement should not be used in the early stage of concrete solidification or in places where a strength of 7 MPa has not been achieved. The rebound number is then too low and the concrete may be damaged [23].
Concrete moisture	Concrete moisture reduces the rebound number by worsening the dynamic hardness.
Measuring place	An appropriate size and number of measuring points are required. According to [24], the minimum size of the measurement site is 50 cm^2^, while according to [8] the size is 900 cm^2^. The standard [24] requires 12 measuring location with 5 measuring points in each of these places. In turn, the standard [8] requires 9 measuring points in each measuring location.
Thickness of the tested element	Thickness should not be less than 10 cm or greater than 20 cm when accessed from one side, greater than 40 cm when accessed from two sides or greater than 60 cm when accessed from three sides [8,25].
Measurement site	Measuring points should be evenly distributed over the surface and be located no less than 3 cm from the edge. They should not be arranged in places where coarse aggregate and reinforcement is less than 3 cm from the surface [26].

**Table 2:** Cementitious mixes used to prepare the samples.

*w/c* Ratio [-]	Mass of Water [kg]	Cement Mass [kg]
0.4	5.0	12.5
0.5	5.5	11.0

**Table 3:** Designation of samples and their characteristics.

Designation of the Sample	*w/c* Ratio	The Number of Layers of Applied Hydrophobic Agent	Type of the Hydrophobic Agent
4	0.4	0	-
41A	0.4	1	A
42A	0.4	2	A
43A	0.4	3	A
41B	0.4	1	B
42B	0.4	2	B
43B	0.4	3	B
5	0.5	0	-
51A	0.5	1	A
52A	0.5	2	A
53A	0.5	3	A
51B	0.5	1	B
52B	0.5	2	B
53B	0.5	3	B

**Table 4:** Estimated strength of cementitious materials impregnated using hydrophobic agents based on near-surface hardness measurements.

Sample	Rebound Number *R* (-)	Estimated Strength *f_R_* (MPa)	The Difference between a Sample with and without Impregnation	
			(MPa)	(%)
4	15.4	45.15	-	-
41A	18.6	46.66	1.52	3.00
42A	20.9	48.33	3.18	7.00
43A	20.4	47.93	2.78	6.00
41B	18.8	46.82	1.67	4.00
42B	20.3	47.81	2.67	6.00
43B	20.2	47.74	2.59	5.00
5	13.9	33.35	-	-
51A	17.4	34.64	1.29	4.00
52A	18.8	35.43	2.08	6.00
53A	16.5	34.21	0.86	3.00
51B	14.6	33.52	0.16	0.00
52B	15.1	33.68	0.33	1.00
53B	17.1	34.47	1.11	0.03

## Data Availability

Results available for review upon request to the authors.
